# Comparison of the Clinical Features and Outcomes of Gallbladder Neuroendocrine Carcinoma with Those of Adenocarcinoma: A Propensity Score-Matched Analysis

**DOI:** 10.3390/cancers13184713

**Published:** 2021-09-21

**Authors:** Min-Young Do, Sung-Ill Jang, Hua-Pyong Kang, Eui-Joo Kim, Kyong-Joo Lee, Go-Eun Park, Su-Jee Lee, Dong-Ki Lee, Sang-Myung Woo, Jae-Hee Cho

**Affiliations:** 1 Department of Internal Medicine, Gangnam Severance Hospital, Yonsei University College of Medicine, Seoul 06273, Korea; dmy24@yuhs.ac (M.-Y.D.); aerojsi@yuhs.ac (S.-I.J.); dklee@yuhs.ac (D.-K.L.); 2 Department of Internal Medicine, Gachon University Gil Medical Center, Incheon 21565, Korea; rabbit9644@gmail.com (H.-P.K.); imetkim@gilhospital.com (E.-J.K.); 3 Department of Internal Medicine, Yonsei University Wonju College of Medicine, Wonju 26426, Korea; smild123@yonsei.ac.kr; 4 Biostatistics Collaboration Unit, Yonsei University College of Medicine, Seoul 06273, Korea; gogoeun@yuhs.ac (G.-E.P.); leeverda@yuhs.ac (S.-J.L.); 5 Center for Liver and Pancreatobiliary Cancer, National Cancer Center, Goyang 10408, Korea

**Keywords:** gallbladder, neuroendocrine neoplasm, neuroendocrine carcinoma, adenocarcinoma, propensity score matching

## Abstract

**Simple Summary:**

Neuroendocrine neoplasms (NENs) of the gallbladder (GB) are extremely rare. We aimed to compare the clinical features of GB-NENs with those of adenocarcinomas (ADCs) of the GB. Among 21 patients with GB-NENs, 20 were diagnosed with poorly differentiated small-cell neuroendocrine carcinoma (NEC), and 1 patient had large-cell NEC. At initial presentation, all patients had advanced stages of cancer, with extensive local extension and/or distant metastasis. Nine patients with GB-NEC who underwent surgical resection had a significantly better progression-free survival (PFS) than those who did not undergo surgery. After a propensity score matching with a 1:1 ratio using the American Joint Committee on Cancer (AJCC) stage, age, sex, and operation status, there was no difference in the overall survival or PFS between AJCC stage-matched patients with GB-NEC or GB-ADC. In conclusion, GB-NEC is difficult to diagnose early and has a prognosis similar to that of GB-ADC.

**Abstract:**

Neuroendocrine neoplasms (NENs) of the gallbladder (GB) are extremely rare. We aimed to compare the clinical features, disease progression, management, and prognosis of patients with GB-NENs with those of patients with GB-adenocarcinomas (ADCs). A total of 21 patients with GB-NENs and 206 patients with GB-ADCs, treated at three tertiary medical centers between January 2010 and December 2020, were enrolled. Of the 21 patients with GB-NENs, 20 were diagnosed with poorly differentiated small-cell neuroendocrine carcinomas (NECs), and 1 patient had large-cell NEC. All patients presented with advanced stages of cancer with extensive local extension and/or distant metastasis and non-specific symptoms. Tumor-node-metastasis stage IIIB and IV (A/B) tumors were found in 6 and 15 (1/14) patients, respectively. Nine patients with GB-NEC who underwent surgical resection had a significantly better progression-free survival (PFS) than those who did not undergo surgery. After a propensity score matching with a 1:1 ratio using the American Joint Committee on Cancer stage, age, sex, and operation status, 19 pairs of patients were included. Compared with stage-matched patients with GB-ADC, patients with GB-NEC had similar overall survival and PFS. However, as GB-NEC is rarely diagnosed early, further studies investigating methods for the early diagnosis and improvement in the survival of patients with GB-NEC are needed.

## 1. Introduction

Neuroendocrine neoplasms (NENs) are a rare malignant disease. According to the National Cancer Institute’s Surveillance, Epidemiology, and End Results (SEER) database, the incidence of NEN is 6.98 per 100,000 people [1,2]. NENs in the gastrointestinal tract are the most common, found in 66% of all NENs. In the gastrointestinal tract, NENs commonly occur in the rectum, ileum, stomach, colon, and pancreas, while gallbladder (GB) NENs are extremely rare [3,4,5]. Previous studies have reported that primary GB-NENs were found in only 0.5% of all NENs and comprised 2% of all GB cancers [6,7].

The first report on GB-NEN was published in 1929 [8]. Since then, small-cell and large-cell carcinomas of the GB have been reported to have neuroendocrine differentiation [9]. According to the World Health Organization (WHO) classification published in 2019, NENs are classified as neuroendocrine tumors (NETs), neuroendocrine carcinomas (NECs), and mixed endocrine non-endocrine neoplasms (MiNENs) [10]. NENs are categorized based on the degree of differentiation, proliferation, and molecular differences. Well-differentiated NENs are categorized as neuroendocrine tumors (NETs), and poorly differentiated NENs are categorized as neuroendocrine carcinomas (NECs) [10].

The pathogenesis and treatment of NENs are being determined rapidly; however, data on GB-NENs are lacking. Unlike symptomatic NENs of the gastrointestinal tract, GB-NENs are asymptomatic and usually found at an aggressive stage [11]. There is no consensus on the treatment strategy for GB-NEN due to its low incidence and unknown course of disease. Currently, the treatment of GB-NEN is similar to that of adenocarcinoma (ADC) of the GB, which is the most common GB cancer. Despite the lack of an established surgical strategy, radical surgical resection is often considered [12,13,14].

We aimed to retrospectively analyze the clinical features, disease progression, management, and prognosis of GB-NENs. In addition, we compared survival outcomes in patients with GB-NENs with those with GB-ADCs using a propensity score-matched cohort.

## 2. Materials and Methods

### 2.1. Patients

Patients with GB-NENs who were treated at three tertiary medical centers (Gangnam Severance Hospital, Gil Medical Center, and National Cancer Center) between January 2010 and December 2020 were included in the study. Data related to these patients were retrospectively collected from electronic medical records.

The inclusion criteria were as follows: (1) the primary tumor site was GB; (2) patients were diagnosed with GB-NENs based on pathology and immunohistochemistry, according to the 2019 WHO classification [10]; and (3) the medical records and data of the patients were accurate and detailed. The exclusion criteria were as follows: (1) MiNENs with an ADC component of ≥30%; (2) patients without detailed and intact the medical records or data. Patients with GB-ADCs were selected for propensity score matching with patients with GB-NENs. Between January 2010 and 21 December 2020 cases of GB-NENs (all patients had G3 NECs) and 206 cases of GB-ADC were included in the study.

### 2.2. Pathological Classification and Staging

The diagnosis of GB-NENs was based on the 2019 WHO classification of tumors of the digestive system. GB-NENs are classified into well-differentiated NETs, poorly differentiated NEC, and MiNENs. GB-NENs are classified as grades 1, 2, and 3 (G1, G2, and G3) based on their mitotic rate and/or the Ki-67 index: G1, mitotic rate of <2 per 2 mm^2^ and/or Ki-67 index of <3%; G2, mitotic rate of 2 to 20 per 2 mm^2^ and/or Ki-67 index of 3 to 20%; and G3, mitotic rate of >20 per 2 mm^2^ and/or Ki-67 index of >20%. GB-NECs are classified as small-cell type or large-cell type. MiNENs consist of a neuroendocrine component or non-neuroendocrine component, such as an adenocarcinoma [10].

The tumor-node-metastasis (TNM) stage of GB neoplasms was determined according to the 8th American Joint Committee on Cancer (AJCC) staging system (Table S1) [15].

### 2.3. Statistical Analysis

Differences between the two groups were compared using the independent two-sample test for continuous variables or the chi-square test for categorical variables. The Kaplan–Meier method was used to evaluate the overall survival outcomes in the two groups. Propensity score values for the predicted probability of survival in patients with GB-NENs and GB-ADCs were estimated using logistic regression analysis, with age, sex, stage, and operation status as confounding variables. Participants with GB-NENs were matched to those with GB-ADC in a 1:1 manner using a nearest-neighbor matching method with a greedy algorithm. All *p*-values were two-sided. Statistical significance was set at *p* < 0.05. Statistical analyses were performed using SPSS software (version 25.0; IBM Corporation, Armonk, NY, USA).

## 3. Results

### 3.1. Clinical Features of GB-NENs

**Figure 1:**
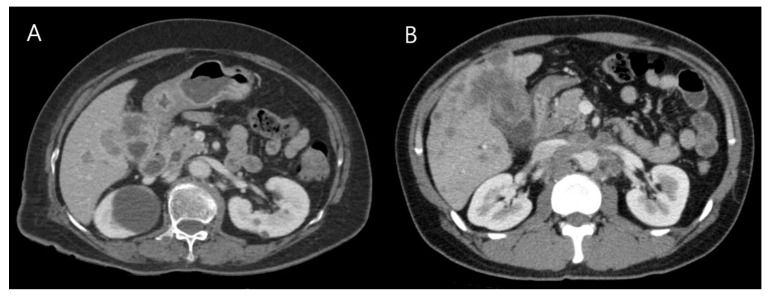
Computed tomography images of gallbladder neuroendocrine carcinoma and adenocarcinoma. (**A**) Gallbladder neuroendocrine carcinoma with direct invasion and metastases to the liver and regional lymph nodes. (**B**) Gallbladder adenocarcinoma with direct invasion and metastases to the liver and regional lymph nodes.

Of the 21 patients, 11 (52.4%) were male (Table 1). The median patient age was 62.0 (range, 31–84) years. The initial clinical presentations were abdominal pain (*n* = 18, 85.8%), jaundice (*n* = 6, 28.6%), weight loss (*n* = 4, 19%), and pruritus (*n* = 1, 4.8%). Serum levels of tumor biomarkers were measured. CA19-9 was elevated in 7 out of the 21 patients, with a median value of 22.6 U/mL (normal value < 37 U/mL), and carcinoembryonic antigen was elevated in 2 out of the 21 patients with values of 1265.3 and 5.6 ng/mL (normal value <5.0 ng/mL). All 21 patients underwent computed tomography (CT), which showed space-occupying lesions in the GB. The findings were similar to those observed in patients with GB-ADCs (Figure 1).

### 3.2. Histopathological Features and Staging

According to the 2019 WHO classification, all patients with GB-NENs were diagnosed with poorly differentiated grade 3 (G3) GB-NECs; 20 patients were diagnosed with small-cell NEC, and 1 patient was diagnosed with large-cell NEC. The Ki-67 index was elevated in 17 patients (>20%). Immunohistochemical staining was used to evaluate NEC biomarkers. Chromogranin A and synaptophysin were positive in 17 (81.0%) and 18 (85.7%) patients, respectively. Positive rates for neuro-specific enolase and CD56 were 100% (tested in 1 patient) and 73.3% (tested in 15 patients), respectively.

At the time of initial diagnosis, all patients presented with advanced cancer with extensive local extension and/or distant metastasis. The number of patients with TNM stages IIIB, IVA, and IVB was 6, 1, and 14, respectively. The liver was the most common site of metastasis in 10 patients (47.6%). Metastases to the bone, peritoneum, ovary, and left subclavian lymph nodes were observed in one (4.8%) case each.

### 3.3. Treatments and Clinical Outcomes

Surgical treatments were performed in 9 of the 21 (42.9%) patients with GB-NEC. Three patients underwent radical cholecystectomy, four patients underwent extended cholecystectomy with hepatic resection, and two patients underwent a simple cholecystectomy for palliative therapy. Postoperative chemotherapy was administered to seven of the nine patients. Among the remaining 12 patients, 8 patients received only chemotherapy, and four patients were not treated. A summary of clinical characteristics of the patients with GB-NEC is shown in Table S2.

**Figure 2:**
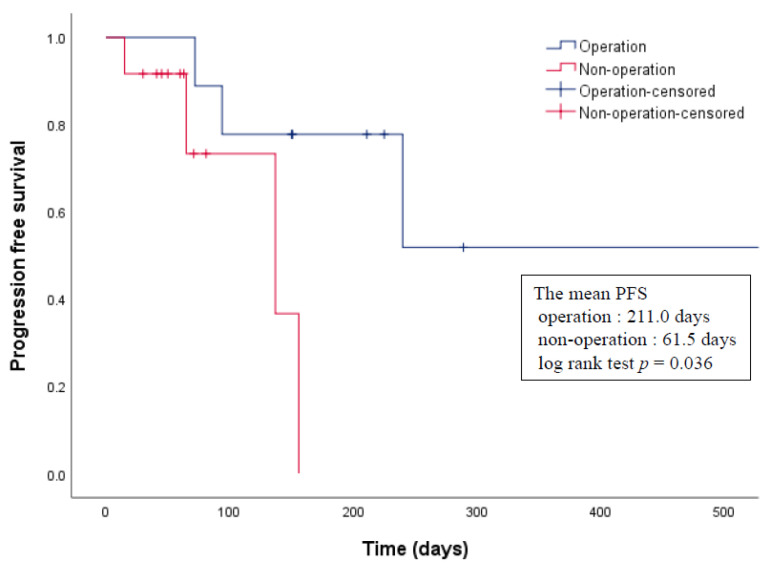
Comparison of the progression-free survival rate between operated and non-operated patients with GB-NEC. GB, gallbladder; NEC, neuroendocrine carcinoma; PFS, progression-free survival.

The median survival was 460 days (range, 15–928 days), and the median progression-free survival (PFS) was 137 days (range, 15–577 days). The median overall survival of operated and non-operated patients was 416.0 days (range, 72–928 days) and 85.5 days (range, 15–348 days), respectively. There was no significant difference between the groups (log rank test *p* = 0.134). The median PFS of operated and non-operated patients was 211.0 days (range, 72–577 days) and 61.5 days (range, 15–156 days), respectively, and the difference was statistically significant (log-rank test, *p* = 0.036) (Figure 2). Univariate survival analysis demonstrated that age and sex had no prognostic significance.

### 3.4. Propensity Score Analysis

The clinical features, treatments and outcomes of 206 patients with GB-ADC are shown in Table S3. Before propensity score matching, the median overall survival of patients with GB-NECs and GB-ADCs was 166 days (range, 15–928 days) and 309 days (range, 19–6552 days). There were no significant differences in overall survival (log rank test *p* = 0.153) (Figure S1). The median PFS of patients with GB-NECs and GB-ADCs was 81 days (range, 15–577 days) and 277 days (range, 19–6552 days), and the difference was statistically significant (log-rank test, *p* < 0.001) (Figure S2). Since the stage was different between patients with GB-NEC and ADC, we conducted propensity score matching.

Propensity score matching for the predicted probability of survival in patients with GB-NECs and GB-ADCs was performed using age, sex, stage, and operation status as confounding variables. After propensity score matching with a 1:1 ratio, 19 pairs of patients were included in this study. In addition, density plot of propensity score is shown in Figure S3.

**Figure 3:**
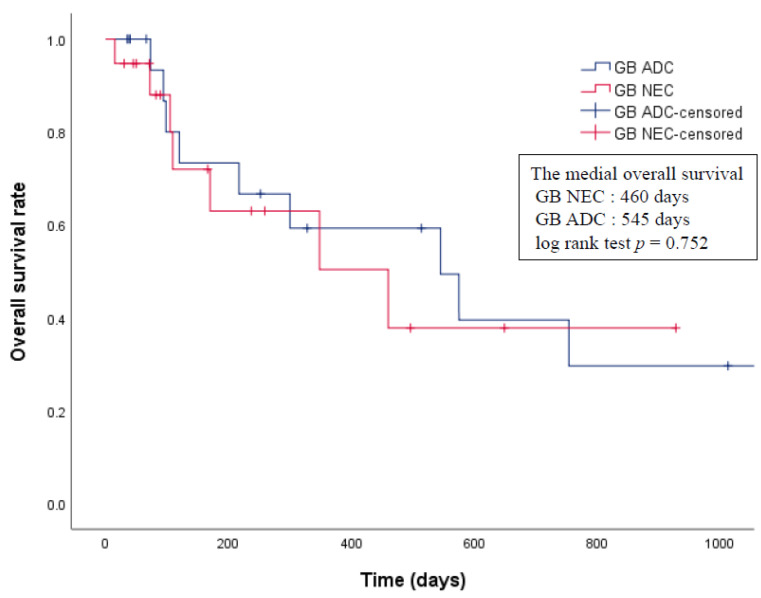
Comparison of the overall survival rate between patients with GB-NEC and GB-ADC. ADC, adenocarcinomas; GB, gallbladder; NEC, neuroendocrine carcinoma.

**Figure 4:**
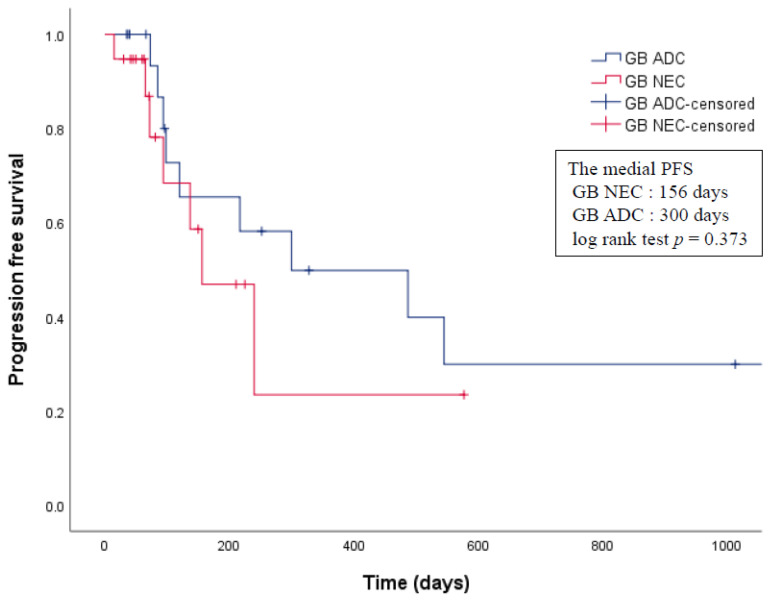
Comparison of the progression-free survival rate between GB-NEC and GB-ADC. ADC, adenocarcinomas; GB, gallbladder; NEC, neuroendocrine carcinoma; PFS, progression-free survival.

The baseline characteristics of the pre- and post-matched groups are presented in Table 2. There were no significant differences between the two matched groups with respect to age, sex, TNM stage, and operation. The median overall survival and median PFS of patients with GB-NECs and GB-ADCs was 460 days (range, 15–928 days) and 545 days (range, 35–2396 days), and 156 days (range, 15–577 days) and 300 days (range, 35–2396 days), respectively. There were no significant differences in the overall survival nor PFS between patients with GB-NECs and those with GB-ADCs after matching the AJCC stages (log rank test *p* = 0.752, *p* = 0.373, respectively) (Figure 3 and Figure 4).

## 4. Discussion

GB-NENs are very rarely encountered in clinical practice [6,16,17]. Therefore, the literature regarding these tumors is largely limited to case reports with literature reviews and a few small-case series. Thus, the assessment of the clinical characteristics and prognosis of patients with GB-NENs is limited. We investigated the clinical features and outcomes of 21 patients with GB-NENs who underwent treatment at three tertiary medical centers between 2010 and 2020. In most previous reports, GB-NENs were confirmed as GB-NECs, and, in this study, all 21 cases were identified as GB-NECs. Therefore, we used propensity score matching for an objective and accurate comparison of the characteristics of GB-NECs with those of GB-ADCs.

GB-NEC is highly malignant with aggressive progression, and systemic metastasis is common, even in the early stages. Most patients are diagnosed at an aggressive stage [17,18,19]. The clinical presentation of GB-NEC is non-specific compared with that of GB-ADC. As most of these cases are non-functional NECs, the presence of carcinoid syndrome is rare [20,21,22]. With the advancements in imaging techniques, imaging examinations including CT, magnetic resonance imaging, and positron emission tomography-CT are helpful for diagnosis of GB diseases. A well-defined margin is the most significant CT finding in GB-NECs compared to GB-ADCs. GB-NECs are mass-replacing tumors with a well-defined margin, while GB-ADCs are thick-walled tumors with poorly defined margins due to their infiltrative nature [23,24,25].

The definite diagnosis of GB-NEC requires pathology and immunohistochemistry. Currently, the most commonly used specific biomarkers are chromogranin A (CgA), synaptophysin (Syn), and neuro-specific enolase [26]. In a previous study involving 15 patients with GB-NECs, CgA and Syn had positive rates of 92.3% and 100%, respectively [11]. In another study, there were eight patients with GB-NEC. CgA and Syn were positive in all cases [27]. In addition, CgA is a soluble secretory glycoprotein secreted by dense-core secretory of neuroendocrine cells [28,29,30], and it has been found that 60% to 80% of NENs in the digestive system contain elevated serum CgA levels [31,32,33]. Serum CgA is related to tumor burden, and the serial measurement of CgA may be useful to detect recurrence [29,34]. In a previous study involving 44 patients with NENs, the sensitivity and specificity of CgA for detecting NEN were 86% and 88%, respectively [31]. In our study, more than 80% of cases showed positive staining for CgA and synaptophysin in immunohistochemical staining. However, during the study period, the introduction and application of serum chromogranin under the Korean National Health Insurance Service was difficult, so the test could not be performed.

According to the statistics of the SEER database, the pathological degree of differentiation of GB-NEC has been reported as well-differentiated tumors (2.4%), moderately differentiated tumors (7.3%), and poorly or undifferentiated tumors (89.7%) [6]. In addition, previous studies have reported that an elevated Ki-67 index and a high mitotic rate are likely to be predictive of a poor prognosis [35,36]. More than 90% of small-cell carcinomas are poorly differentiated, with regional or distant metastasis at diagnosis [37]. In our study, all 21 patients with GB-NECs showed poorly differentiated tumors, with Ki-67 index >20, and small-cell type NECs, which were seen in 20 patients, were predominant.

In general, the only curative therapeutic modality for GB cancer is surgical resection. Moreover, because extensive debulking resection is preferred for gastroenteropancreatic NENs, surgical resection is one of the most important treatments for GB-NECs. Although a surgical treatment strategy for GB-NECs has not been established, previous studies have recommended aggressive radical cholecystectomy, including lymph node dissection and/or hepatic resection. A previous study showed that patients who underwent surgery had better survival than those who did not [12,13,14]. In the SEER database, patients with GB-NENs who underwent GB surgery had better survival than patients who did not [1]. Radical resection has a significantly better survival outcome than non-operative palliative treatment [12]. In contrast, the outcome after palliative resection is not significantly different from that after non-operative palliative treatment [12]. In a previous study involving 15 patients with GB-NECs, radical cholecystectomy was performed in 10 patients. The overall survival after surgical treatment was not significantly longer than that after non-surgical treatment [11]. In the present study, nine patients underwent surgical treatment, including radical cholecystectomy and simple cholecystectomy, and all patients classified as TNM stage III underwent radical cholecystectomy. Similar to the results of previous studies, patients with GB-NEC who underwent surgery had a significantly better PFS than those who underwent non-surgical treatment.

The prognosis of patients with GB-NECs is reported to be relatively worse compared with that of patients with GB-ADCs. In a retrospective analysis of 10 patients with GB-NECs, the median survival was 3.0 months, and the one-, two-, and three-year cumulative survival rates were 20%, 10%, and 0%, respectively. In contrast, the median survival in 377 patients with GB-ADCs treated during the same period was 6.0 months, and the one-, two-, three-, and 5-year survival rates were 38.0%, 31.0%, 30.1%, and 28.4%, respectively [17]. In a study of 15 propensity-matched pairs of patients with GB-NECs and ADCs, patients with GB-NECs had a worse overall survival rate than those with GB-ADCs [11]. However, this small retrospective study included five patients with MiNEC among 15 patients with GB-NECs. Yun et al. [13] compared the overall five-year survival rate between four patients with GB-NECs and 38 patients with GB-ADCs and reported no significant differences between the two groups. Interestingly, these results were similar to our findings that there was no difference in prognosis between GB-NEC and GB-ADC among patients matched according to their AJCC stage, which is the strongest prognostic factor [19]. In the preliminary comparison between the GB-NEC and GB-ADC cohorts, it was confirmed that GB-NEC is difficult to diagnose early, and most cases are diagnosed at an advanced stage. The reasons for this are as follows: GB-ADC is diagnosed at various stages because, in some patients, it is incidentally diagnosed early, following laparoscopic cholecystectomy for the removal of gallstones or polyps [9,38]. However, as GB-NEC is highly malignant and progresses aggressively, most patients are diagnosed at a late stage, when the opportunity for surgical treatment has been lost. Moreover, early diagnosis is difficult due to the lack of research into the pathophysiology of GB-NEC. Several studies have suggested that adjuvant chemotherapy is needed at an advanced stage of GB-NEC, even if radical resection is completed. Adjuvant chemotherapy is the treatment of choice when radical resection is not feasible [27,39,40,41]. In our study, 15 out of the 21 patients received chemotherapy; however, the chemotherapy regimen was inconsistent.

There are several limitations to our study. First, this was a retrospective study, and there were only 21 patients with GB-NEC. Due to the insufficient number of patients, we could not validate statistical comparisons by multivariate analysis. However, we conducted statistical comparisons using propensity score matching and compared the outcomes of patients with GB-NEC with those of patients with GB-ADC. Second, to compare the typical characteristics of GB-NEC, MiNENs were excluded from the analysis. However, because many GB-NECs were diagnosed as MiNENs, their characteristics could not be confirmed in this study. Third, accurate values of serum CgA and exact percentage of the Ki-67 index were not confirmed due to the limitations of the retrospective study. Lastly, the heterogeneity of treatments including various chemotherapeutic agents and surgery might have influenced the therapeutic outcomes and prognosis of patients with GB-NEC.

Our propensity matching analysis indicated that patients with GB-NEC had prognoses similar to those with GB-ADC in the matched AJCC stage. In addition, GB-NEN is asymptomatic, and usually found in an advanced stage of GB-NEC. The univariate analysis showed that surgery helped progression-free survival: patients who underwent surgery showed better progression-free survival than those who did not. Further studies investigating the early diagnosis and improvement in the survival of GB-NEC are needed [42].

Due to the lack of research on the mechanism of occurrence and treatment, further laboratory and clinical studies with large sample sizes are needed to establish early diagnosis and improvement in the survival of those with GB-NENs.

## 5. Conclusions

GB-NECs are poorly differentiated tumors that have non-specific symptoms, and they are diagnosed at an advanced clinical stage. Pathological and immunohistochemical evaluations are required to confirm the diagnosis. Aggressive radical cholecystectomy is the preferred treatment option. Although uncertain, chemotherapy is critical for the management of unresectable GB-NEC. The AJCC stage-based propensity-score-matched analysis showed that patients with GB-ADC had an overall survival and PFS similar to patients with GB-NEC. Further studies investigating the early diagnosis and improvement in the survival of patients with GB-NEC are needed.

## Figures and Tables

**Table 1:** Clinical features of 21 cases of gallbladder neuroendocrine carcinoma.

Variables	Median (Range) or *n* (%)
Age, years, median (range)	62.0 (31–84)
Sex, *n* (%)	
Male	11 (52.4)
Female	10 (47.6)
Clinical symptom, *n* (%)	
Abdominal discomfort	18 (85.8)
Jaundice	6 (28.6)
Weight loss	4 (19.0)
Pruritus	1 (4.8)
Tumor marker, median (range)	
CA19-9 (U/mL)	22.6 (0.6–696.7)
CEA (ng/mL)	2.2 (0.5–1265.3)
NEN classification, *n* (%)	
NEC, small-cell type	20 (95.2)
NEC, large-cell type	1 (4.8)
Differentiated degree, *n* (%)	
Grade 1/2	0/0
Grade 3	21 (100)
Ki-67 index, *n* (%)	
>20	17 (81.0)
Unknown	4 (19.0)
Immunohistochemical stain, *n* (%)	
CgA	17 (81.0)
Syn	18 (85.7)
NSE (test in 1 case)	1 (100)
CD56 (test in 15 cases)	11 (73.3)
TNM stage, *n* (%)	
IIIB	6 (28.6)
IVA	1 (4.8)
IVB	14 (66.7)
Distant metastasis, *n* (%)	13 (61.9)
Liver	11
Bone	1
Peritoneum	1
Ovary	1
Lt. subclavian lymph node	1
Operation, *n* (%)	9 (42.9)
Curative	7
Palliative	2
Chemotherapy, *n* (%)	15 (71.4)

CA19-9, carbohydrate antigen 19-9; CEA, carcinoembryonic antigen; CgA, chromogranin A; NEC, neuroendocrine carcinoma; NEN, neuroendocrine neoplasm; NSE, neuron-specific enolase; Syn, synaptophysin; TNM, tumor node metastasis.

**Table 2:** Clinical characteristics of gallbladder neuroendocrine carcinoma and gallbladder adenocarcinoma (before and after propensity score matching).

Variables	before PS Matching				after PS Matching			
	GB-NEC (*n* = 21)	GB-ADC (*n* = 206)	Standardized Difference	*p*-Value	GB-NEC (*n* = 19)	GB-ADC (*n* = 19)	Standardized Difference	*p*-Value
Age, years, mean (SD)	64.2 ± 14.3	65.0 ± 11.1	0.059	0.774	64.6 ± 15.1	63.1 ± 13.8	−0.102	0.755
Sex, *n* (%)				0.471				0.746
Male	11 (52.4)	91(44.2)	−0.165		9 (47.4)	10 (52.6)	0.105	
Female	10 (47.6)	115 (55.8)	0.165		10 (52.6)	9 (47.4)	−0.105	
TNM stage, *n* (%)				0.011				>0.999
I	0 (0.0)	17 (8.3)	0.424		0 (0.0)	0 (0.0)	0.000	
II	0 (0.0)	32 (15.5)	0.606		0 (0.0)	0 (0.0)	0.000	
IIIa	0 (0.0)	10 (4.9)	0.319		0 (0.0)	0 (0.0)	0.000	
IIIb	6 (28.6)	15 (7.3)	−0.578		4 (21.1)	4 (21.1)	0.000	
IVa	1 (4.8)	6 (2.9)	−0.096		1 (5.3)	2 (10.5)	0.196	
IVb	14 (66.7)	126 (61.2)	−0.115		14 (73.7)	13 (68.4)	−0.116	
Operation, *n* (%)	9 (42.9)	115 (55.8)	0.262	0.255	7 (36.8)	9 (47.4)	0.214	0.511
Chemotherapy, *n* (%)	15 (71.4)	141 (68.4)	−0.065	0.779	13 (68.4)	14 (73.7)	0.116	0.721

ADC, adenocarcinoma; GB, gallbladder; NEC, neuroendocrine carcinoma; PS, propensity score; TNM, tumor node metastasis. Data are presented as mean (SD) or number (%). *p*-values are calculated by independent two sample *t-*test for continuous variables or Chi-squared test for categorical variables.

## Data Availability

The data used in this study are available in this article.

## Supplementary Materials

The following are available online at https://www.mdpi.com/article/10.3390/cancers13184713/s1, Figure S1: Comparison of the overall survival rate between patients with GB-NEC and GB-ADC before PS matching, Figure S2: Comparison of the progression-free survival rate between GB-NEC and GB-ADC before PS matching, Figure S3: Density plot of propensity score, Table S1: Gallbladder cancer staging in the 8th American Joint Committee on Cancer, Table S2: Summary of clinical characteristics of the patients with gallbladder neuroendocrine carcinoma, Table S3: Clinical features, treatments and outcomes of 206 patients with gallbladder adenocarcinoma.
