# Supplementary material for: Comparison of the Clinical Features and Outcomes of Gallbladder Neuroendocrine Carcinoma with Those of Adenocarcinoma: A Propensity Score-Matched Analysis
Source: Cancers (Basel). 2021 Sep 21;13(18):4713. doi: 10.3390/cancers13184713 (PMC8471353; doi:10.3390/cancers13184713)
Supplement: Supplementary file 1 [file cancers-13-04713-s001.zip › cancers-1363573-supplementary.pdf]

Supplementary Materials: Comparison of the Clinical Features and Outcomes of Gallbladder Neuroendocrine Carcinoma with Those of Adenocarcinoma: A Propensity Score-Matched Analysis

Min-Young Do, Sung-Ill Jang, Hua-Pyong Kang, Eui-Joo Kim, Kyong-Joo Lee, Go-Eun Park, Su-Jee Lee, Dong-Ki Lee, Sang-Myung Woo and Jae-Hee Cho

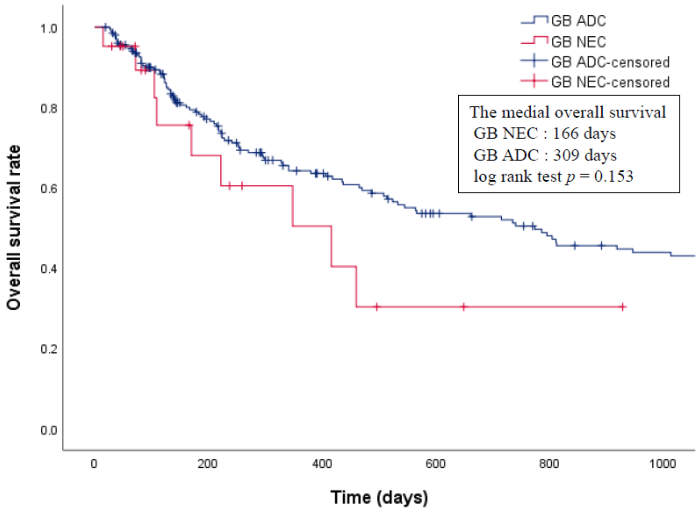

Figure S1. Comparison of the overall survival rate between patients with GB-NEC and GB-ADC before PS matching.

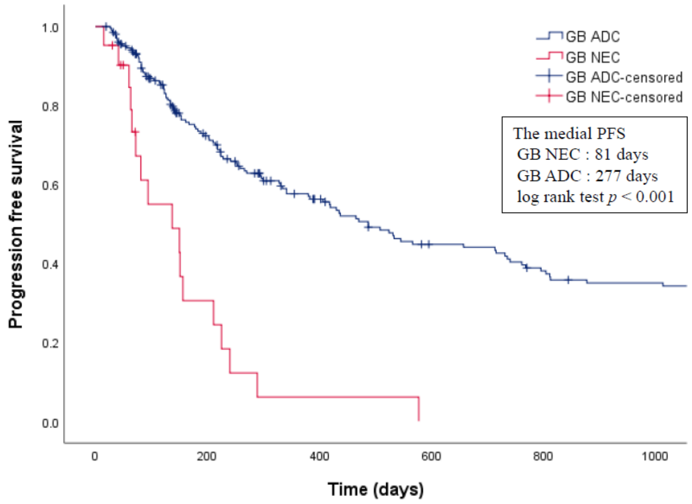

Figure S2. Comparison of the progression-free survival rate between GB-NEC and GB-ADC before PS matching.

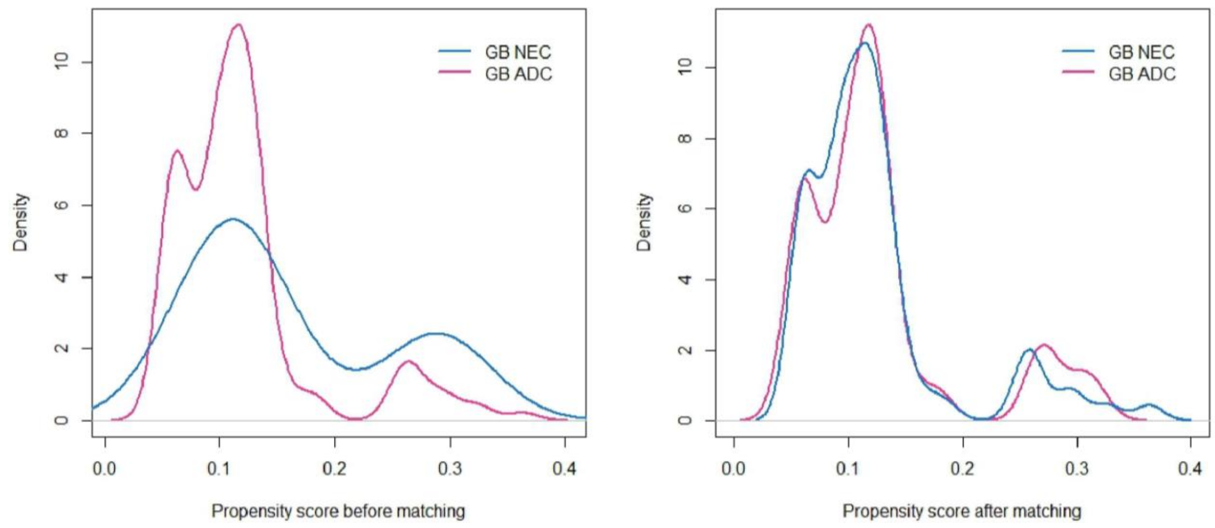

Figure S3. Density plot of propensity score.

Table S1. Gallbladder cancer staging in the 8th American Joint Committee on Cancer [15].

Tumor	Definition
Tis	Carcinoma in situ
T1	Tumor invades the lamina propria or muscular layer
T1a	Invades the lamina propria
T1b	Invades the muscle layer
T2	Invades the perimuscular connective tissue
T2a	Invades the perimuscular connective tissue on the peritoneal side
T2b	Invades the perimuscular connective tissue on the hepatic side
T3	Perforates the serosa and/or directly invades the liver and/or other adjacent organs or structures (stomach, duodenum, colon, pancreas, omentum, or extrahepatic bile ducts)
T4	Invades the main portal vein or hepatic artery or two or more extrahepatic organs or structures
Node	
N0	No regional lymph node metastasis
N1	Metastasis in 1–3 regional lymph nodes
N2	Metastasis in ≥ 4 regional lymph nodes
Metastasis	
M0	No distant metastasis
M1	Distant metastasis present
Stage	
0	TisN0M0
I	T1N0M0
IIA	T2aN0M0
IIB	T2bN0M0
IIIA	T3N0M0
IIIB	T1N1M0, T2N1M0, T3N1M0
IVA	T4N0M0, T4N1M0
IVB	Any T, N2M0<br>Any T, Any N, M1

Table S2. Summary of clinical characteristics of the patients with gallbladder neuroendocrine carcinoma.

No.	Age (Year)	Sex	Symptoms	Size (mm)	TNM Grade	Distant Metastasis	WHO Classifcation	Ki-67 (%)	IHC	Operation	Operation Purpose	Chemo-Therapy Regimen	PFS (Day)	Overall Survival (Day)
1	74	F	Abdominal pain, jaundice	NA	IVA		NEC, small cell type	> 20	CgA (+), Syn (+), CD56 (+)			EP	81	259
2	45	M	Jaundice	78	IVB	Liver	NEC, small cell type	> 20	CgA (+), Syn (+)	Extended cholecystectomy with Rt. Hepatectomy	Curative	EP	211	496
3	74	F	Abdominal pain, abdominal mass	20	IIIB		NEC, large cell type	> 20	CgA (+), Syn (+), NSE (+), CD56 (+)	Laparoscopic cholecystectomy	Palliative		72	72
4	59	M	Abdominal pain	NA	IVB	Liver	NEC, small cell type	> 20	CgA (+), Syn (+)			EP	30	30
5	48	F	Abdominal pain, weight loss	NA	IVB	Liver	NEC, small cell type	NA	CgA (−), Syn (−), CD56 (+)			EP	63	166
6	62	M	Abdominal pain	40	IIIB		NEC, small cell type	> 20	CgA (+), Syn (+)	Radical cholecystectomy	Curative	EP	289	416
7	79	F	Abdominal pain	50	IVB	Liver	NEC, small cell type	> 20	CgA (+), Syn (+)	Extended cholecystectomy and Lt. hepatectomy	Curative		577	928
8	57	F	Abdominal pain	32	IIIB		NEC, small cell type	> 20	CgA (+), Syn (+)	Radical cholecystectomy	Curative	EP	150	649
9	84	F	Abdominal pain	24	IVB	Lt supraclavicular lymph node	NEC, small cell type	> 20	CgA (+), Syn (+)				60	89
10	60	M	Abdominal pain	35	IIIB		NEC, small cell type	NA	CgA (−), Syn (−)	Extended cholecystectomy and S4b/S5 bisegmentectomy	Curative	EP	151	222
11	31	F	Abdominal pain	50	IVB	Peritoneum	NEC, small cell type	> 20	CgA (+), Syn (+)	Laparoscopic cholecystectomy	Palliative	EP	94	105

12	78	M	Health check-up	40	IIIB		NEC, small cell type	> 20	CgA (+), Syn (+)	Extended cholecystectomy and liver segmentectomy	Curative	EP	240	460
13	75	M	Health check-up	38	IIIB		NEC, small cell type	> 20	CgA (+), Syn (+)	Radical cholecystectomy	Curative	EP	225	237
14	68	M	Abdominal pain, jaundice, weight loss	140	IVB	Liver	NEC, small cell type	> 20	CgA (+), Syn (+)				45	45
15	82	M	Abdominal pain, weight loss	47	IVB		NEC, small cell type	NA	CgA (−), Syn (+)				137	170
16	44	M	Abdominal pain, abdominal mass	43	IVB	Liver	NEC, small cell type	> 20	CgA (+), Syn (+), CD56 (+)			EP	65	109
17	66	M	Abdominal pain, jaundice	180	IVB	Liver	NEC, small cell type	> 20	CgA (+), Syn (+), CD56 (+)				15	15
18	57	F	Abdominal pain, jaundice, weight loss	59	IVB	Liver	NEC, small cell type	NA	CgA (−), Syn (+), CD56 (+)			GP	50	50
19	62	M	Abdominal pain	66	IVB	Liver	NEC, small cell type	> 20	CgA (+), Syn (−), CD56 (+)			GP	41	82
20	61	F	Abdominal pain, jaundice, pruritus	35	IVB	Liver	NEC, small cell type	> 20	CgA (+), Syn (+), CD56 (+)			GP	156	348
21	83	F	Abdominal pain, back pain	40	IVB	Liver, Rt. Ovary, L5 vertebra	NEC, small cell type	> 20	CgA (+), Syn (+), CD56 (+)				71	71

CgA, chromogranin A; EP, Etoposide + Cisplatin; F, female; GP, Gemcitabine + Cisplatin; IHC, immunohistochemistry; M, male; NA, not available; NEC, neuroendocrine carcinoma; PFS, progression free survival; Syn, synaptophysin; TMN, tumor node metastasis; WHO, World Health Organization.

Table S3. Clinical features, treatments and outcomes of 206 patients with gallbladder adenocarcinoma.

**Variables**	**Mean (SD) or *n* (%)**
Age, years, mean (SD)	65.00 ± 11.1
Sex, *n* (%)	
Male	91 (44.2)
Female	115 (55.8)
Tumor marker, mean (SD)	
CA19-9 (U/mL)	1924.9 ± 4620.9
CEA (ng/mL)	48.0 ± 237.1
Differentiated degree, *n* (%)	
Well differentiated	29 (14.1)
Moderate differentiated	71 (34.5)
Poorly differentiated	38 (18.4)
Unknown	68 (33.0)
TNM stage, *n* (%)	
I	17 (8.3)
IIA	32 (15.5)
IIB	10 (4.9)
IIIB	15 (7.3)
IVA	6 (2.9)
IVB	126 (61.2)
Distant metastasis, *n* (%)	
Liver	93 (45.1)
Lung	14 (6.8)
Peritoneum	42 (20.4)
Operation, *n* (%)	115 (55.8)
Laparoscopic cholecystectomy	40
Radical cholecystectomy	75
Chemotherapy, *n* (%)	141 (68.4)
Progression free survival, days, mean (SD)	710.3 ± 1010.0
Overall Survival, days, mean (SD)	796.4 ± 1061.9

CA19-9, carbohydrate antigen 19-9; CEA, carcinoembryonic antigen; SD, standard deviation; TMN, tumor node metastasis.
